# Supplementary material for: Serotype Distribution of Remaining Pneumococcal Meningitis in the Mature PCV10/13 Period: Findings from the PSERENADE Project
Source: Microorganisms. 2021 Apr 1;9(4):738. doi: 10.3390/microorganisms9040738 (PMC8066874; doi:10.3390/microorganisms9040738)
Supplement: Supplementary file 1 [file microorganisms-09-00738-s001.pdf]

Serotype Distribution of Remaining Pneumococcal Meningitis in the Mature PCV10/13 Period: Findings from the PSERENADE Project

Table of Contents

Table S1. Percentage of pneumococcal meningitis in the mature PCV10/13 period due to serotypes included in current and upcoming PCV products 2

Table S2. Serotype specific distribution of CSF+ pneumococcal meningitis in the mature PCV10/13 period, by age group 3

Figure S1. Distribution of CSF+ meningitis cases and non-CSF+ clinically-defined meningitis cases in the mature PCV10/13 period by age group 6

Figure S2. Serotype specific distribution of pneumococcal meningitis in the mature PCV10/13 period, including all meningitis cases (CSF+ and non-CSF+ clinically defined meningitis cases) . 7

Figure S3. Modeled distribution for the percentage of CSF+ pneumococcal meningitis due to serotypes included in current and upcoming products, in both PCV10 and PCV13 using sites ... 7

Table S3. Modeled percentage of CSF+ pneumococcal meningitis due to serotypes included in current and upcoming pneumococcal vaccine products, by age group 8

Figure S4. Serotype-specific distribution of CSF+ pneumococcal meningitis in the mature PCV10/13 period, comparing Brazil to the other PCV10-using sites 9

Figure S5. Distribution of top 10 ranking serotypes causing CSF+ pneumococcal meningitis in each PCV13-using site 10

Table S4. Characteristics of sites contributing pneumococcal meningitis cases to analyses. 11

Table S1. Percentage of pneumococcal meningitis in the mature PCV10/13 period due to serotypes included in current and upcoming PCV products

A. CSF+ cases only

Age category	Serotype Category	*PCV13-using sites*	*PCV10-using sites*	
		**(N = 4,444) Mean % (CI)**	**Brazil (N=917) Mean % (CI)**	**Other sites (N=224) Mean % (CI)**
< 5 years	PCV10	7.4 (4.9, 9.5)	4.8 (2.3, 8.6)	4.9 (0.6, 16.5)
	PCV13	14.1 (10.4, 16.2)	40.0 (33.3, 47.0)	29.3 (16.1, 45.5)
	PCV15	20.9 (19.2, 24.3)	41.4 (34.7, 48.4)	40.5 (24.8, 57.9)
	PCV20	56.1 (50.5, 60.1)	61.4 (54.5, 68.1)	53.9 (37.2, 69.9)
	PCV24	60.4 (54.2, 65.2)	63.8 (56.9, 70.3)	56.4 (39.6, 72.2)
	PPV23	59.8 (54.2, 64.6)	61.0 (54.0, 67.6)	56.4 (39.6, 72.2)
≥ 5 years	PCV10	9.8 (7.5, 11.8)	7.6 (5.8, 9.9)	14.9 (10.1, 21.0)
	PCV13	25.8 (23.6, 27.6)	38.5 (34.9, 42.2)	37.0 (30.0, 44.5)
	PCV15	31.8 (29.3, 34.1)	40.6 (37.0, 44.3)	39.1 (31.4, 47.2)
	PCV20	57.0 (54.3, 61.9)	53.3 (49.6, 57.1)	63.5 (55.4, 71.0)
	PCV24	63.4 (60.5, 69.2)	57.9 (54.1, 61.5)	69.2 (61.4, 76.4)
	PPV23	62.8 (60.1, 69.0)	56.3 (52.6, 60.0)	68.0 (60.0, 75.2)

B. All meningitis cases (CSF+ and non-CSF+ clinically defined meningitis)

Age category	Serotype Category	*PCV13-using sites*	*PCV10-using sites*	
		**(N = 5,353) Mean % (CI)**	**Brazil (N=1,020) Mean % (CI)**	**Other sites (N=240) Mean % (CI)**
< 5 years	PCV10	7.1 (5.9, 10.6)	5.1 (2.7, 8.7)	4.9 (0.6, 16.5)
	PCV13	14.8 (10.7, 17.5)	40.3 (33.9, 46.8)	29.3 (16.1, 45.5)
	PCV15	22.6 (19.6, 24.0)	41.5 (35.2, 48.1)	40.5 (24.8, 57.9)
	PCV20	56.6 (52.3, 63.1)	61.0 (54.5, 67.3)	53.9 (37.2, 69.9)
	PCV24	61.4 (56.2, 66.1)	63.6 (57.1, 69.7)	56.4 (39.6, 72.2)
	PPV23	60.8 (55.3, 65.5)	60.6 (54.1, 66.9)	56.4 (39.6, 72.2)
≥ 5 years	PCV10	9.2 (7.7, 10.5)	7.5 (5.8, 9.6)	13.7 (9.2, 19.3)
	PCV13	25.2 (22.8, 26.5)	37.8 (34.4, 41.3)	34.0 (27.4, 41.1)
	PCV15	30.8 (28.2, 32.1)	39.8 (36.4, 43.3)	38.4 (31.1, 46.1)
	PCV20	57.2 (53.3, 59.5)	53.1 (49.5, 56.6)	64.0 (56.3, 71.1)
	PCV24	63.4 (60.0, 66.2)	57.8 (54.2, 61.3)	69.8 (62.3, 76.5)
	PPV23	62.7 (59.0, 65.7)	56.1 (52.6, 59.6)	68.6 (61.1, 75.5)

Serotypes included in each product: PCV7 (4, 6B, 9V, 14, 18C, 19F, 23F), PCV10 (PCV7 + 1, 5, 7F), PCV13 (PCV10 + 3, 6A, 19A), PCV15 (PCV13 + 22F, 33F), PCV20 (PCV15 + 8, 10A, 11A, 12F, 15BC), PCV24 (PCV20 + 2, 9N, 17F, 20), PPV23 (PCV24 - 6A). PCV13 is Pfizer's Prevnar13/Prevenar13; PCV10 is GSK's Synflorix.

Table S2. Serotype specific distribution of CSF+ pneumococcal meningitis in the mature PCV10/13 period, by age group

A. Children < 5 years of age

Serotype	*PCV13-using sites* (N=1,173) Mean % (CI)	*PCV10-using sites* Combined (N=243) Mean % (CI)	Brazil (N=210) Mean % (CI)	Other sites (N=32) Mean % (CI)
15BC	11.5 (8.0, 15.1)	6.6 (3.8, 10.5)	7.1 (4.0, 11.5)	3.1 (0.1, 16.2)
10A	7.1 (4.1, 10.2)	4.5 (2.3, 8.0)	4.8 (2.3, 8.6)	3.1 (0.1, 16.2)
8	7.1 (2.5, 11.8)	4.1 (2.0, 7.5)	3.8 (1.7, 7.4)	6.2 (0.8, 20.8)
24F	5.9 (1.1, 10.6)	5.4 (2.9, 9.0)	5.2 (2.6, 9.2)	6.2 (0.8, 20.8)
22F	4.9 (3.0, 6.7)	0.8 (0.1, 3.0)	0.9 (0.1, 3.4)	--
23B	4.8 (3.0, 6.5)	4.1 (2.0, 7.5)	3.8 (1.7, 7.4)	6.2 (0.8, 20.8)
12F	4.6 (2.2, 7.0)	2.1 (0.7, 4.8)	1.4 (0.3, 4.1)	6.2 (0.8, 20.8)
3	4.0 (2.1, 5.8)	7.4 (4.5, 11.5)	8.1 (4.8, 12.6)	3.1 (0.1, 16.2)
33F	3.9 (1.8, 6.0)	2.1 (0.7, 4.8)	0.5 (0.0, 2.6)	12.5 (3.5, 29.0)
15A	3.8 (2.2, 5.5)	1.2 (0.3, 3.6)	0.9 (0.1, 3.4)	3.1 (0.1, 16.2)
19F	3.6 (1.6, 5.5)	--	--	--
35B	3.1 (1.6, 4.5)	2.5 (0.9, 5.3)	2.9 (1.1, 6.1)	--
27	2.6 (0.8, 4.4)	--	--	--
19A	2.5 (1.5, 3.6)	24.4 (19.1, 30.3)	24.3 (18.6, 30.7)	25.0 (11.5, 43.4)
16F	2.3 (0.8, 3.8)	2.9 (1.2, 5.9)	3.3 (1.4, 6.8)	--
9N	1.9 (0.7, 3.1)	0.8 (0.1, 3.0)	0.9 (0.1, 3.4)	--
38	1.9 (0.7, 3.0)	0.4 (0.0, 2.3)	--	3.1 (0.1, 16.2)
21	1.8 (0.8, 2.8)	0.4 (0.0, 2.3)	--	3.1 (0.1, 16.2)
35F	1.7 (0.5, 2.8)	0.4 (0.0, 2.3)	0.5 (0.0, 2.6)	--
23A	1.4 (0.5, 2.4)	2.5 (0.9, 5.3)	2.9 (1.1, 6.1)	--
17F	1.3 (0.6, 2.1)	0.8 (0.1, 3.0)	0.9 (0.1, 3.4)	--
11A	1.2 (0.2, 2.2)	2.5 (0.9, 5.3)	2.9 (1.1, 6.1)	--
20	1.2 (1.0, 1.5)	0.4 (0.0, 2.3)	0.5 (0.0, 2.6)	--
6C	1.0 (0.2, 1.8)	10.3 (6.8, 14.9)	10.0 (6.3, 14.9)	12.5 (3.5, 29.0)
34	0.7 (0.5, 1.0)	0.4 (0.0, 2.3)	0.5 (0.0, 2.6)	--
13	0.6 (0.1, 1.2)	0.8 (0.1, 3.0)	0.9 (0.1, 3.4)	--
14	0.6 (0.0, 1.3)	--	--	--
1	0.6 (0.4, 0.8)	--	--	--
6A	--	2.5 (0.9, 5.3)	2.9 (1.1, 6.1)	--
25A	--	2.1 (0.7, 4.8)	2.4 (0.8, 5.5)	--
7F	--	2.1 (0.7, 4.8)	2.4 (0.8, 5.5)	--
23F	--	0.8 (0.1, 3.0)	0.9 (0.1, 3.4)	--
28A	--	0.8 (0.1, 3.0)	0.9 (0.1, 3.4)	--
15F	--	0.4 (0.0, 2.3)	0.5 (0.0, 2.6)	--
18B	--	0.4 (0.0, 2.3)	0.5 (0.0, 2.6)	--
18C	--	0.4 (0.0, 2.3)	0.5 (0.0, 2.6)	--
29	--	0.4 (0.0, 2.3)	0.5 (0.0, 2.6)	--
6B	--	0.4 (0.0, 2.3)	0.5 (0.0, 2.6)	--
9V	--	0.4 (0.0, 2.3)	0.5 (0.0, 2.6)	--
36	--	0.4 (0.0, 2.3)	0.5 (0.0, 2.6)	--
10B	--	0.4 (0.0, 2.3)	--	3.1 (0.1, 16.2)
31	--	0.4 (0.0, 2.3)	--	3.1 (0.1, 16.2)

B. Persons ≥ 5 years of age.

Serotype	*PCV13-using sites*	*PCV10-using sites*		
	**(N=3,271) Mean % (CI)**	**Combined (N=860) Mean % (CI)**	**Brazil (N=706) Mean % (CI)**	**Other sites (N=154) Mean % (CI)**
3	13.1 (8.8, 17.4)	13.9 (11.7, 16.4)	14.9 (12.3, 17.7)	9.7 (5.5, 15.6)
8	8.0 (4.3, 11.6)	2.9 (1.9, 4.3)	2.3 (1.3, 3.6)	5.8 (2.7, 10.8)
12F	6.3 (3.6, 8.9)	4.6 (3.3, 6.3)	3.5 (2.3, 5.2)	9.7 (5.5, 15.6)
23B	5.6 (3.7, 7.6)	5.3 (3.9, 7.1)	5.0 (3.5, 6.8)	7.1 (3.6, 12.4)
23A	4.8 (2.7, 6.9)	6.3 (4.8, 8.1)	7.1 (5.3, 9.2)	2.6 (0.7, 6.5)
22F	4.8 (3.4, 6.1)	2.3 (1.4, 3.6)	1.8 (1.0, 3.1)	4.5 (1.8, 9.1)
10A	4.4 (3.4, 5.5)	2.2 (1.3, 3.4)	1.7 (0.9, 3.0)	4.5 (1.8, 9.1)
9N	4.2 (2.8, 5.5)	2.4 (1.5, 3.7)	2.0 (1.1, 3.3)	4.5 (1.8, 9.1)
19F	4.0 (2.2, 5.9)	1.5 (0.8, 2.6)	1.1 (0.5, 2.2)	3.2 (1.1, 7.4)
15BC	3.9 (2.9, 4.9)	2.7 (1.7, 4.0)	2.7 (1.6, 4.2)	2.6 (0.7, 6.5)
15A	3.8 (2.1, 5.4)	3.1 (2.1, 4.5)	3.7 (2.4, 5.3)	0.6 (0.0, 3.6)
19A	3.1 (1.9, 4.3)	13.3 (11.1, 15.7)	14.4 (11.9, 17.3)	7.8 (4.1, 13.2)
35F	2.9 (1.6, 4.2)	0.8 (0.3, 1.7)	0.8 (0.3, 1.8)	0.6 (0.0, 3.6)
11A	2.8 (1.5, 4.0)	2.4 (1.5, 3.7)	2.5 (1.5, 4.0)	1.9 (0.4, 5.6)
16F	2.4 (1.1, 3.8)	1.9 (1.1, 3.0)	1.8 (1.0, 3.1)	1.9 (0.4, 5.6)
6C	2.2 (1.2, 3.3)	10.1 (8.2, 12.3)	10.1 (7.9, 12.5)	10.4 (6.1, 16.3)
24F	1.9 (0.7, 3.1)	1.5 (0.8, 2.6)	1.6 (0.8, 2.8)	1.3 (0.2, 4.6)
35B	1.7 (0.6, 2.9)	2.0 (1.2, 3.1)	2.0 (1.1, 3.3)	1.9 (0.4, 5.6)
33F	1.6 (0.8, 2.5)	0.5 (0.1, 1.2)	0.3 (0.0, 1.0)	1.3 (0.2, 4.6)
31	1.3 (0.6, 2.0)	0.1 (0.0, 0.6)	--	0.6 (0.0, 3.6)
17F	1.2 (0.7, 1.8)	0.9 (0.4, 1.8)	1.0 (0.4, 2.0)	0.6 (0.0, 3.6)
20	1.1 (0.9, 1.3)	1.4 (0.7, 2.4)	1.6 (0.8, 2.8)	0.6 (0.0, 3.6)
23F	1.0 (0.4, 1.6)	1.2 (0.6, 2.1)	1.3 (0.6, 2.4)	0.6 (0.0, 3.6)
7C	1.0 (0.2, 1.7)	0.7 (0.3, 1.5)	0.8 (0.3, 1.8)	--
14	0.8 (0.4, 1.2)	1.3 (0.6, 2.3)	1.0 (0.4, 2.0)	2.6 (0.7, 6.5)
7F	0.8 (0.4, 1.2)	0.8 (0.3, 1.7)	0.6 (0.1, 1.4)	1.9 (0.4, 5.6)
13	0.6 (0.4, 0.7)	1.7 (1.0, 2.9)	2.0 (1.1, 3.3)	0.6 (0.0, 3.6)
1	0.5 (0.4, 0.6)	0.2 (0.0, 0.8)	0.1 (0.0, 0.8)	0.6 (0.0, 3.6)
4	--	2.1 (1.2, 3.3)	2.0 (1.1, 3.3)	2.6 (0.7, 6.5)
34	--	1.5 (0.8, 2.6)	1.8 (1.0, 3.1)	--
6A	--	1.5 (0.8, 2.6)	1.6 (0.8, 2.8)	1.3 (0.2, 4.6)
25A	--	1.2 (0.6, 2.1)	1.4 (0.7, 2.6)	--
18C	--	1.2 (0.6, 2.1)	1.1 (0.5, 2.2)	1.3 (0.2, 4.6)
28A	--	0.8 (0.3, 1.7)	0.8 (0.3, 1.8)	0.6 (0.0, 3.6)
18A	--	0.7 (0.3, 1.5)	0.8 (0.3, 1.8)	--
21	--	0.3 (0.1, 1.0)	0.3 (0.0, 1.0)	0.6 (0.0, 3.6)
9V	--	0.3 (0.1, 1.0)	0.3 (0.0, 1.0)	0.6 (0.0, 3.6)
11B	--	0.2 (0.0, 0.8)	0.3 (0.0, 1.0)	--
15F	--	0.2 (0.0, 0.8)	0.3 (0.0, 1.0)	--
29	--	0.2 (0.0, 0.8)	0.3 (0.0, 1.0)	--
35C	--	0.2 (0.0, 0.8)	0.3 (0.0, 1.0)	--
6B	--	0.2 (0.0, 0.8)	0.1 (0.0, 0.8)	0.6 (0.0, 3.6)
18B	--	0.1 (0.0, 0.6)	0.1 (0.0, 0.8)	--
19C	--	0.1 (0.0, 0.6)	0.1 (0.0, 0.8)	--
24A	--	0.1 (0.0, 0.6)	0.1 (0.0, 0.8)	--
36	--	0.1 (0.0, 0.6)	0.1 (0.0, 0.8)	--
19B	--	0.1 (0.0, 0.6)	0.1 (0.0, 0.8)	--
6D	--	0.1 (0.0, 0.6)	0.1 (0.0, 0.8)	--

10F	--	0.1 (0.0, 0.6)	--	0.6 (0.0, 3.6)
25F	--	0.1 (0.0, 0.6)	--	0.6 (0.0, 3.6)

Serotypes are ordered by percentage in PCV13-using sites (modeled output) then by percentage in PCV10-using sites (pooled output). Values shown here correspond to Figure 4, with the added distribution for Brazil and other PCV10-using sites shown separately. For PCV13-using sites, empty values correspond to serotypes that were not modeled because of their low ranking in a pooled distribution. For PCV10-using sites, empty values correspond to serotypes that had zero cases. PCV13 is Pfizer's Prevnar13/Prevenar13; PCV10 is GSK's Synflorix.

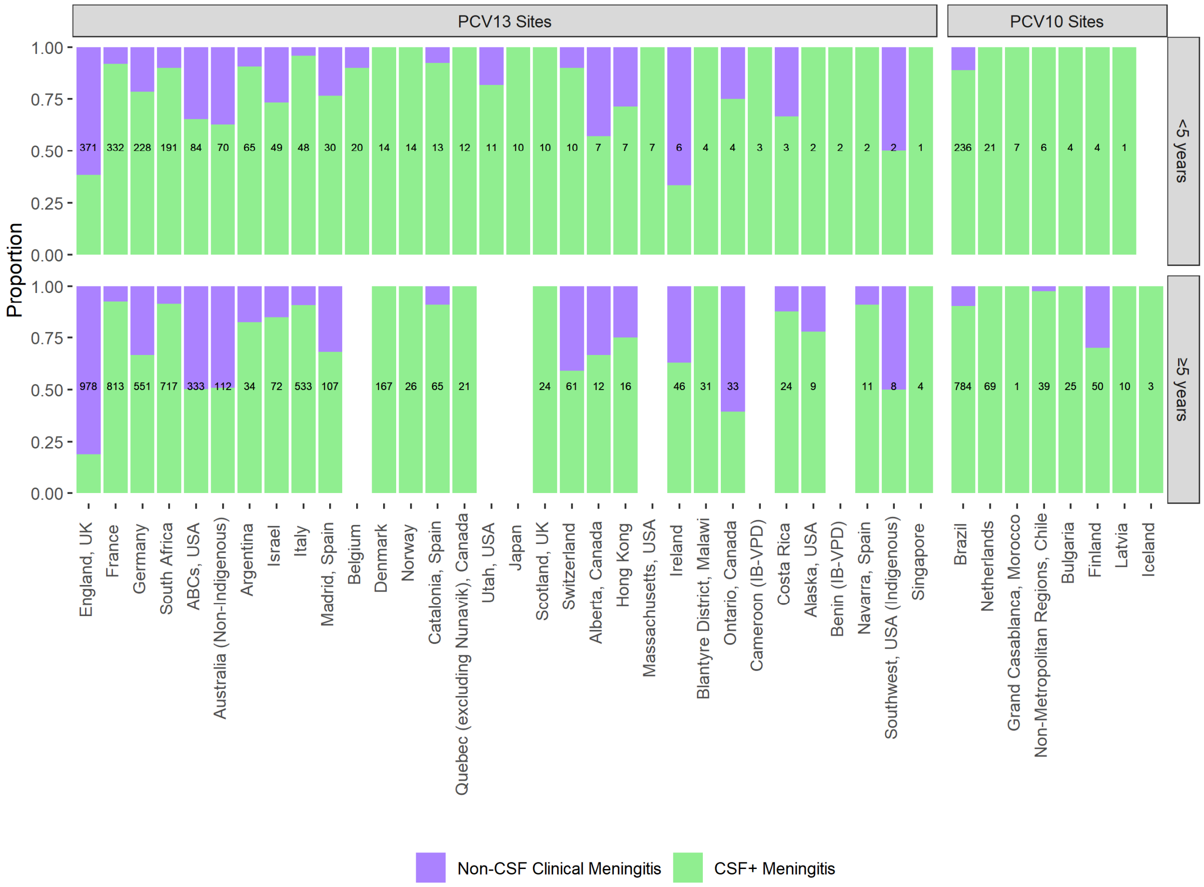

Figure S1. Distribution of CSF+ meningitis cases and non-CSF+ clinically-defined meningitis cases in the mature PCV10/13 period by age group. Sites are ordered by decreasing number of cases within PCV product used/ age group stratum (number in bar). PCV13 is Pfizer's Prevnar13/Prevenar13; PCV10 is GSK's Synflorix.

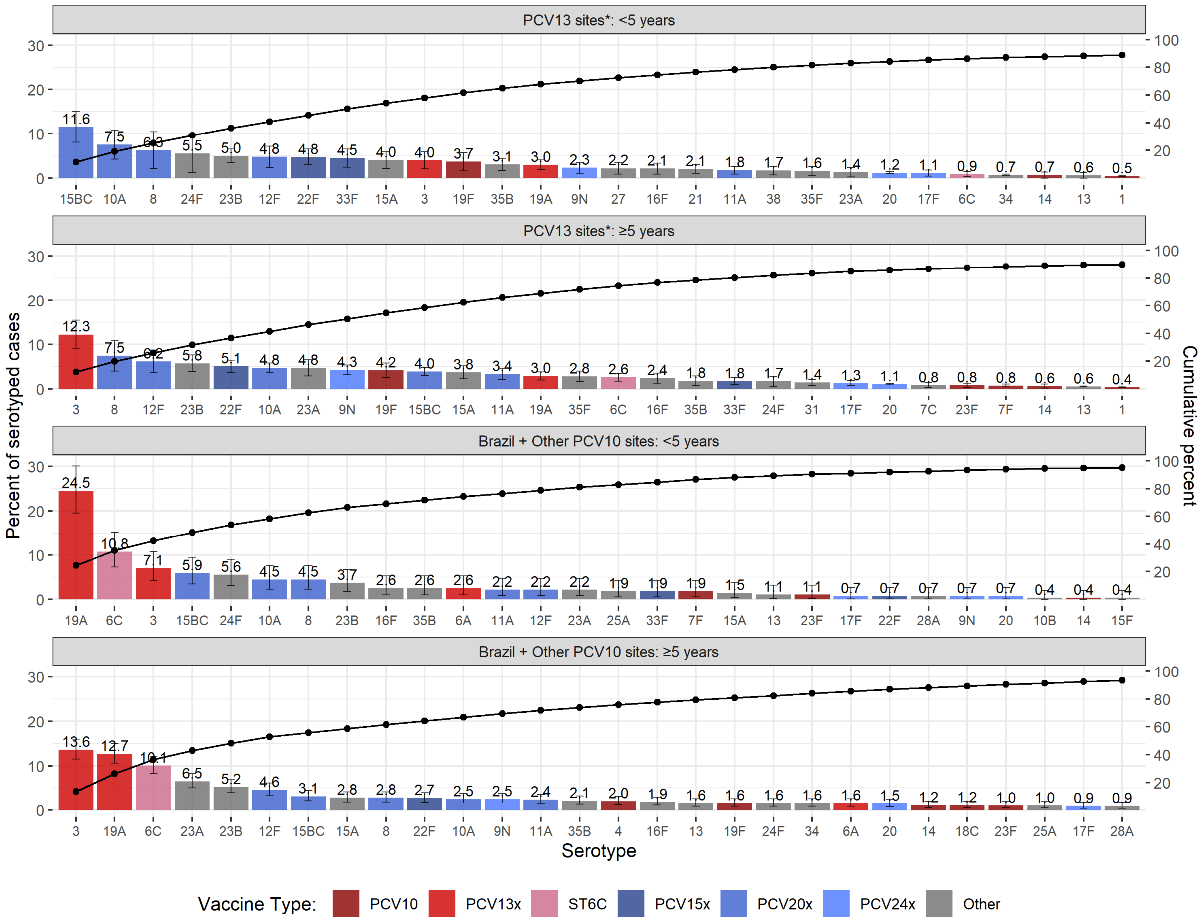

Figure S2. Serotype specific distribution of pneumococcal meningitis in the mature PCV10/13 period, including all meningitis cases (CSF+ and non-CSF+ clinically defined meningitis cases). Serotypes are colored by the lowest valency PCV product they are included in. The "x" in the PCV legend represents the additional serotypes included in that product relative to the next lower product (i.e., PCV13x includes serotypes 3, 6A, and 19A). PCV13 is Pfizer's Prevnar13/Prevenar13; PCV10 is GSK's Synflorix. Serotype 6C is colored separately because although it is not included in any product it covered through cross-protection with 6A [1]. Morocco and Bulgaria were not included in the PCV10 distribution due to serotyping limitations. *Serotype distribution for PCV13 sites is modeled output for the top 25 serotypes based on a pooled ranking plus serotype 1. Serotype distribution for PCV10 sites is from pooling cases across sites.

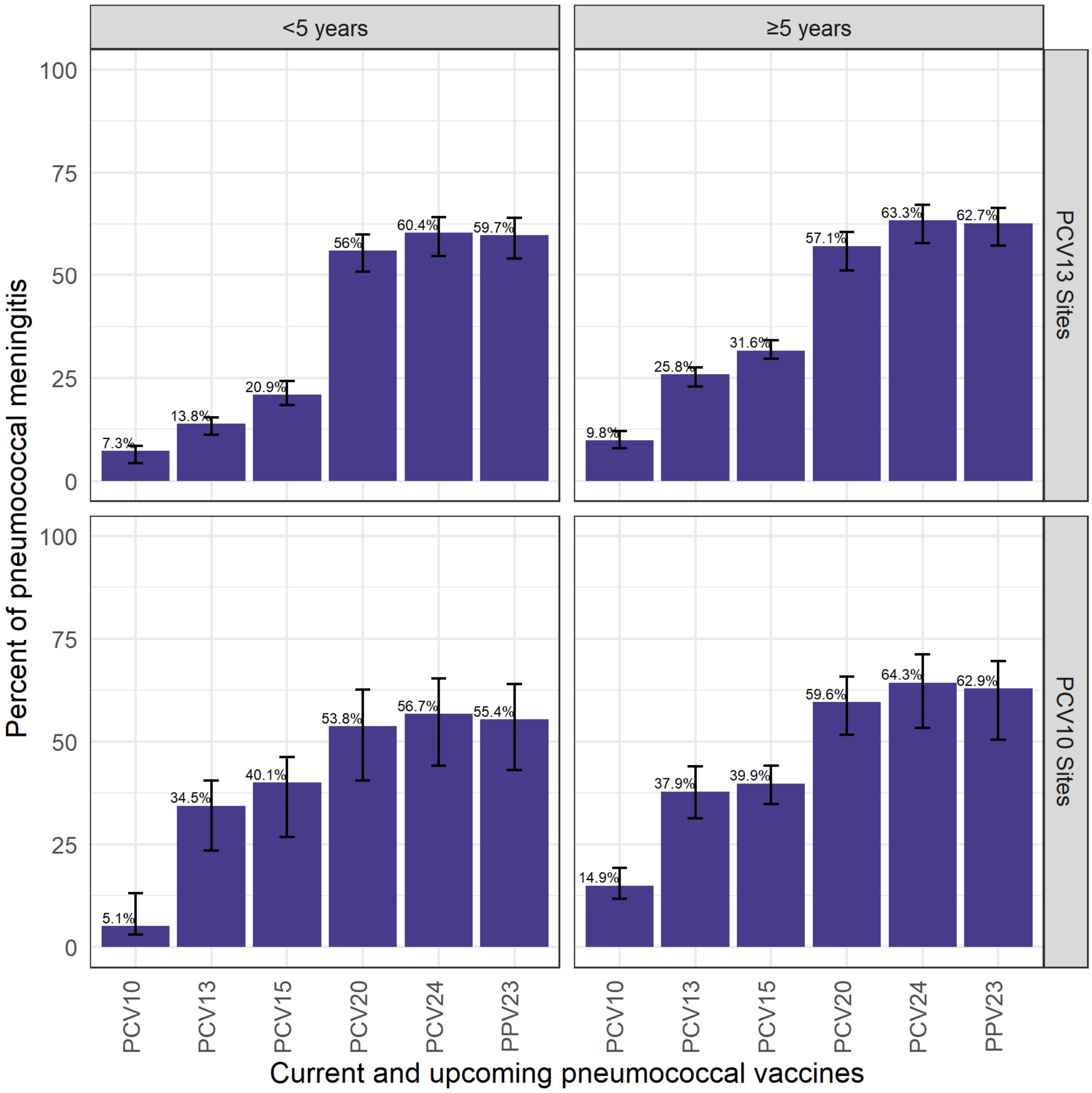

Figure S3. Modeled distribution for the percentage of CSF+ pneumococcal meningitis due to serotypes included in current and upcoming products, in both PCV10 and PCV13 using sites. The model includes all sites regardless of PCV product in use and uses a covariate for product to produce PCV−stratified results. Separate models were run by age group. The modeled output accounts for heterogeneity between the sites and uncertainty from low case counts (conveyed by width of confidence intervals). Results for PCV10−using sites should not be interpreted as representative of all PCV10-using sites globally given the limited data. Serotypes included in each product: PCV7 (4, 6B, 9V, 14, 18C, 19F, 23F), PCV10 (PCV7 + 1, 5, 7F), PCV13 (PCV10 + 3, 6A, 19A), PCV15 (PCV13 + 22F, 33F), PCV20 (PCV15 + 8, 10A, 11A, 12F, 15BC), PCV24 (PCV20 + 2, 9N, 17F, 20), PPV23 (PCV24 - 6A). PCV13 is Pfizer's Prevnar13/Prevenar13; PCV10 is GSK's Synflorix.

Table S3. Modeled[1] percentage of CSF+ pneumococcal meningitis due to serotypes included in current and upcoming pneumococcal vaccine products, by age group**.**

PCV Product in use	Serotype Category[2]	≥ 5 years Mean % (CI)	≥ 50 years Mean % (CI)	≥ 65 years Mean % (CI)
PCV13	PCV10[3]	9.8 (7.9, 12.0)	8.4 (6.7, 12.4)	7.7 (5.9, 10.3)
	PCV13	25.8 (22.9, 27.6)	24.1 (22.5, 26.7)	25.2 (23.4, 28.6)
	PCV15	31.6 (29.7, 33.2)	31.6 (28.6, 33.7)	33.0 (31.8, 34.9)
	PCV20	57.1 (51.1, 60.5)	56.9 (53.8, 64.0)	56.4 (53.9, 60.1)
	PCV24	63.3 (57.8, 67.1)	63.7 (60.0, 70.5)	64.9 (62.5, 68.6)
	PPV23	62.7 (57.2, 66.5)	63.4 (58.2, 69.0)	64.7 (62.2, 68.4)
PCV10	PCV10[3]	14.9(11.7, 19.2)	12.7 (7.7, 17.2)	15.0 (7.5, 25.7)
	PCV13	37.9(31.4, 44.1)	38.5 (31.9, 44.2)	44.7 (38.0, 52.0)
	PCV15	39.9 (34.8, 44.2)	41.1 (37.9, 47.6)	45.0 (42.7, 50.2)
	PCV20	59.6 (41.6, 65.7)	61.2 (51.2, 67.2)	61.2 (56.7, 66.3)
	PCV24	64.3(53.4, 71.2)	65.4 (52.7, 72.8)	67.1 (60.8, 76.5)
	PPV23	62.9 (50.4, 69.6)	63.8 (50.6, 72.2)	65.0 (57.9, 75.7)

[1]The model includes all sites regardless of PCV product in use and uses a covariate for product to produce PCV-stratified results. Separate models were run by age group. The modeled output accounts for heterogeneity between the sites and uncertainty from low case counts (conveyed by width of confidence intervals). Results for PCV10-using sites should not be interpreted as representative of all PCV10-using sites globally given the limited data. [2]Serotypes included in each product: PCV7 (4, 6B, 9V, 14, 18C, 19F, 23F), PCV10 (PCV7 + 1, 5, 7F), PCV13 (PCV10 + 3, 6A, 19A), PCV15 (PCV13 + 22F, 33F), PCV20 (PCV15 + 8, 10A, 11A, 12F, 15BC), PCV24 (PCV20 + 2, 9N, 17F, 20), PPV23 (PCV24 - 6A). PCV13 is Pfizer's Prevnar13/Prevenar13; PCV10 is GSK's Synflorix.

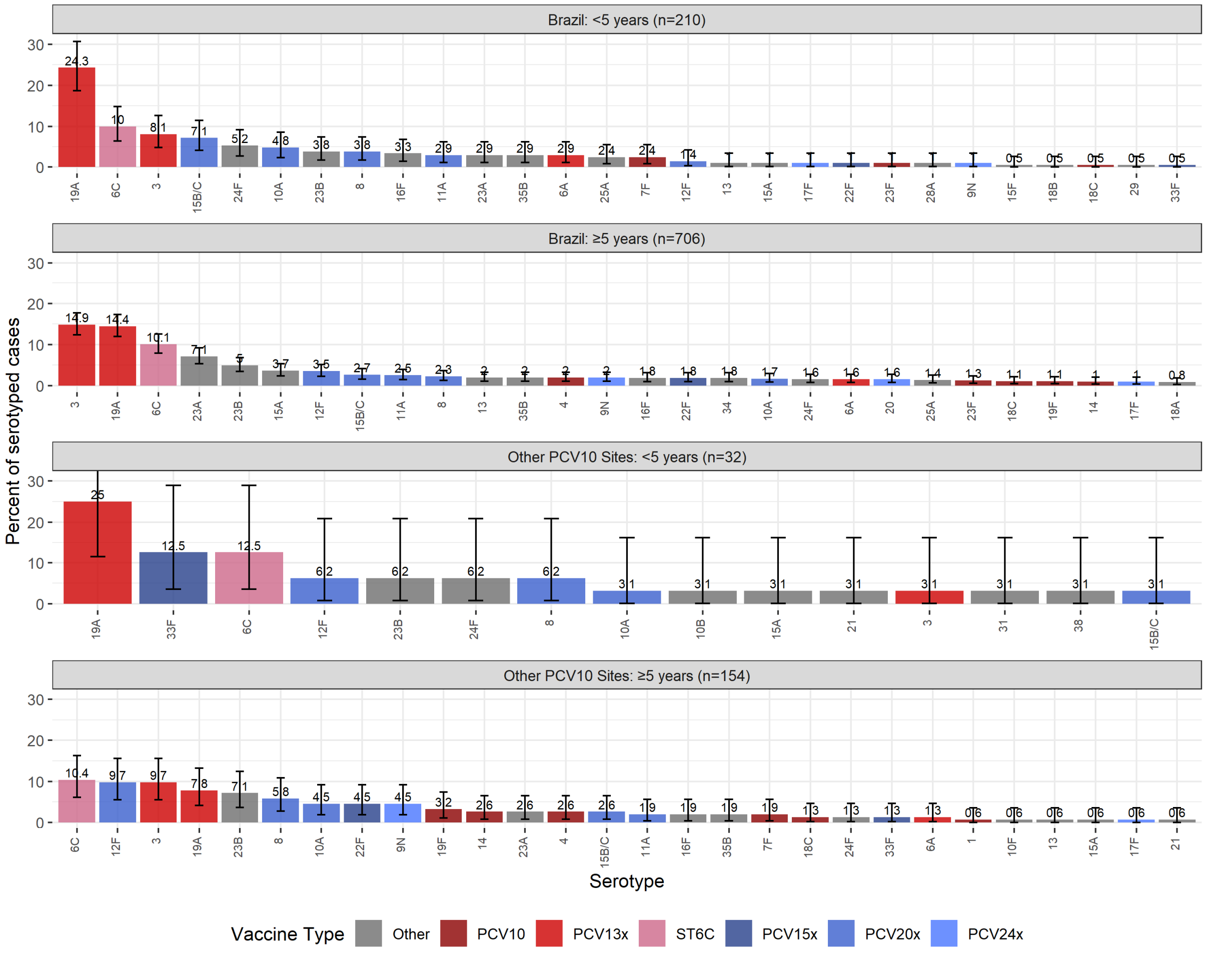

Figure S4. Serotype-specific distribution of CSF+ pneumococcal meningitis in the mature PCV10/13 period, comparing Brazil to the other PCV10-using sites[1]. [1]PCV10 sites include those that had the ability to serotype most non−PCV24−type serotypes (Morocco and Bulgaria excluded). Serotypes are colored by the lowest valency PCV product they are included in. Red/pink hues represent PCV10/13 vaccine-types or vaccine-related-types; blue hues represent serotypes covered by PCV products in development; grey represents non-vaccine types. The “x” in the PCV legend represents the additional serotypes included in that product relative to the next lower product (i.e., PCV13x includes serotypes 3, 6A, and 19A). PCV13 is Pfizer’s Prevnar13/Prevenar13; PCV10 is GSK’s Synflorix. Serotype 6C is colored separately because although it is not included in any product it is covered through cross-protection with PCV13-type serotype 6A [1].

A. Children < 5 years

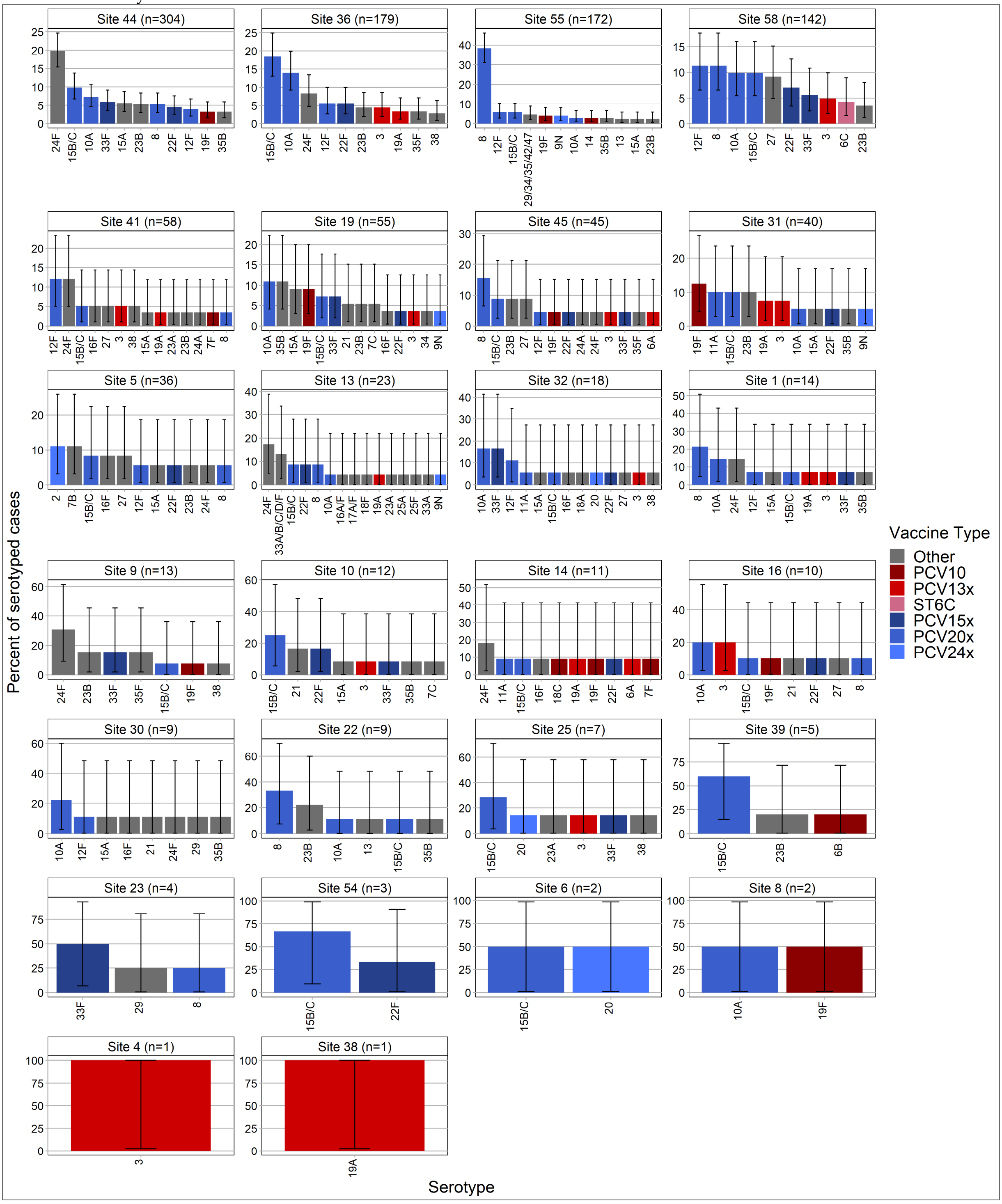

B. Persons ≥ 5 years

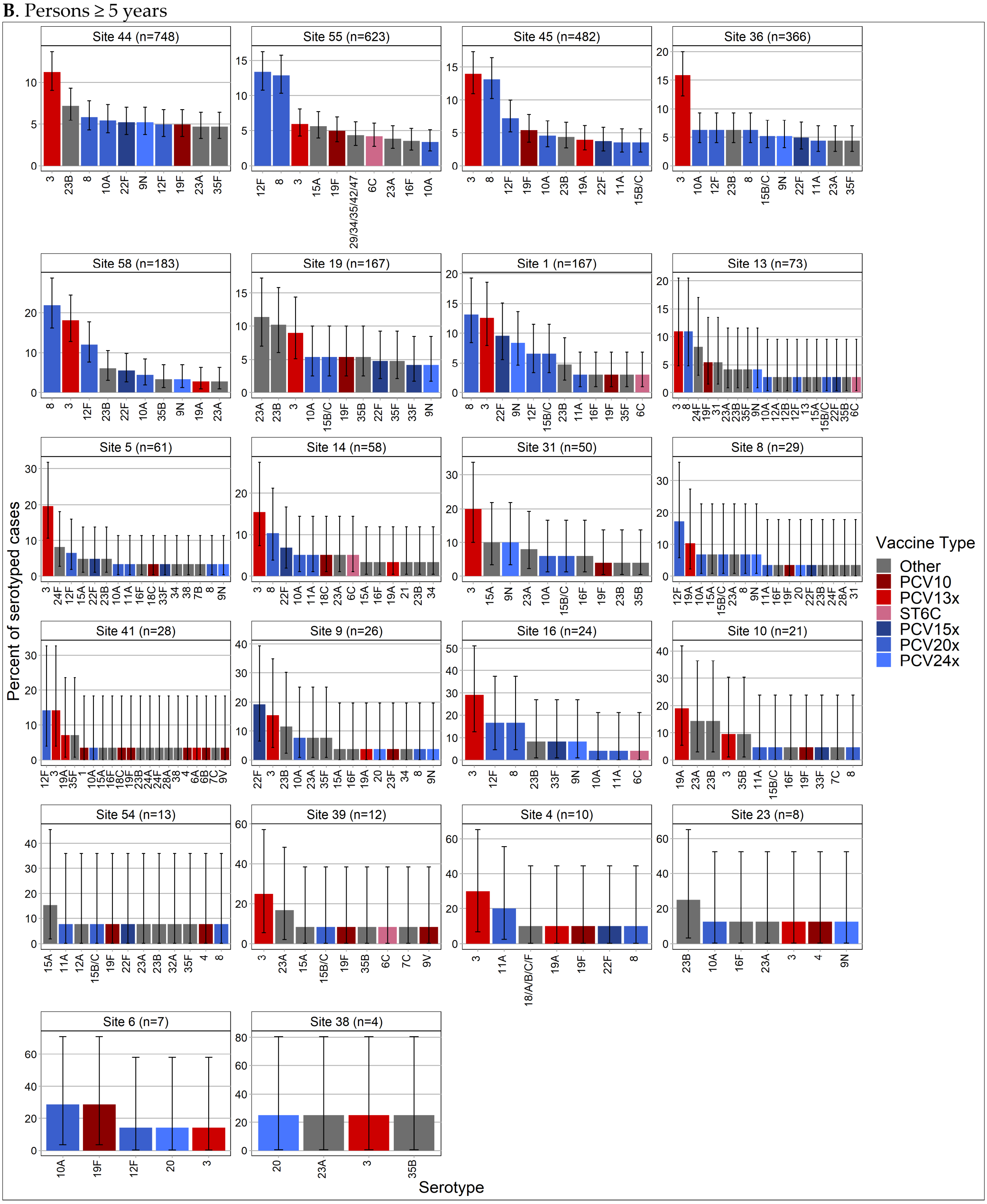

Figure S5. Distribution of top 10 ranking serotypes causing CSF+ pneumococcal meningitis in each PCV13-using site[1-]
[1]Includes sites with the ability to serotype most non-PCV24-type serotypes. Sites are ordered by decreasing number of cases. If multiple serotypes shared rank 10, these were all shown. PCV13 is Pfizer's Prevnar13/Prevenar13; PCV10 is GSK's Synflorix.

Table S4. Characteristics of sites contributing pneumococcal meningitis cases to analyses.

PCV Product[1]	Region	Site	Meningitis definition for cases that did not have pneumococcus detected in CSF[2]	Analytic year of PCV10/13 introduction[3]	Number of mature years in analysis		Serotyping methods[4]
					< 5 years	≥ 5 years	
PCV10	Europe	Bulgaria	*Only CSF+ cases reported*	2010	2	3	Q, L, PCR37
		Finland	Spn in blood & diagnosis from ICD-10 codes	2010	2	2	WGS
		Iceland	Spn in blood & abnormal CSF	2011	2	2	L, PCR
		Latvia	*Only CSF+ cases reported*	2012	1	1	Q, L, CST
		Netherlands	*Only CSF+ cases reported*	2011	4	2	Q, L
	Latin America	Brazil	Spn in blood & (abnormal CSF or diagnosis from ICD-10 codes)	2010	4	3	Q, L, PCR70
		Non-Metropolitan Regions, Chile	Spn in blood & clinical diagnosis	2011	1	1	Q
	Northern Africa & Western Asia	Casablanca, Morocco	Spn in blood & abnormal CSF	2012	1	1	Q, L, PCR70
PCV13	Europe	Belgium	Spn in blood & clinical diagnosis (not from ICD-10 codes)	2011	1	0	Q
		Denmark	*Only CSF+ cases reported*	2010	3	3	Q, L, PCR70
		France	Spn in blood & abnormal CSF	2010	5	3	Q, L, PCR70
		Germany	Spn in blood & clinical diagnosis	2009	5	3	Q, PCR70
		Ireland	Spn in blood & (abnormal CSF or clinical diagnosis (not from ICD-10 codes))	2010	2	2	Q, L, PCR
		Italy	Spn in blood & clinical diagnosis (not from ICD-10 codes)	2010	5	3	Q, L
		Norway	Spn in blood & clinical diagnosis (not from ICD-10 codes)	2011	3	1	Q, NGS
		Catalonia, Spain	Spn in blood & abnormal CSF	2010	1	1	Q, PCR37
		Madrid, Spain	Spn in blood & abnormal CSF	2010	5	3	Q, L, PCR76
		Navarra, Spain	Spn in blood & abnormal CSF	2010	2	2	Q, PCR76
		Switzerland	Spn in blood & (abnormal CSF or clinical diagnosis (not from ICD-10 codes))	2011	3	1	Q
		England, UK	Spn in blood & clinical diagnosis	2010	6	4	Q, L
		Scotland, UK	Spn in blood & clinical diagnosis (not from ICD-10 codes)	2010	5	3	L

North America	Alberta, Canada	Spn in blood & abnormal CSF	2010	4	2	Q
	Ontario, Canada	Spn in blood & (abnormal CSF or diagnosis (not from ICD-10 codes))	2011	3	2	Q
	Quebec (excluding Nunavik), Canada	Spn in blood & clinical diagnosis	2011	3	1	Q
	ABCs, USA	Spn in blood & clinical diagnosis (not from ICD-10 codes)	2010	4	2	Q, PCR70, WGS
	Alaska, USA	Spn in blood & clinical diagnosis	2010	4	2	Q, L, PCR70
	Massachusetts, USA	*Only CSF+ cases reported*	2010	4	2	Q
	Southwest, USA (Indigenous)	Spn in blood & clinical diagnosis	2010	5	3	Q
	Utah	Spn in blood & abnormal CSF	2010	4	0	Q
Latin America	Argentina	Spn in blood & abnormal CSF or clinical diagnosis	2012	2	1	Q, PCR37, PCR70
	Costa Rica	Spn in blood & clinical diagnosis	2011	2	2	Q
Sub-Saharan Africa	Benin (WHO IB-VPD)	*Only CSF+ cases reported*	2011	1	0	PCR
	Blantyre District, Malawi	*Only CSF+ cases reported*	2012	1	1	L, PCR37
	Cameroon (WHO IB-VPD)	*Only CSF+ cases reported*	2011	1	0	PCR
	South Africa	Spn in blood & clinical diagnosis	2011	3	2	Q, PCR38
Northern Africa & Western Asia	Israel	Spn in blood & abnormal CSF	2010	2	2	Q
Asia	Hong Kong	Spn in blood & (abnormal CSF or diagnosis (not from ICD-10 codes))	2011	4	2	PCR35
	Japan	Spn in blood & clinical diagnosis (not from ICD-10 codes)	2014	1	0	Q, PCR70
	Singapore	*Only CSF+ cases reported*	2012	2	2	Q
Oceania	Australia (Non-Indigenous)	Spn in blood & abnormal CSF	2011	3	1	Q, PCR

[1]PCV product that was used in the analysis (which met the mature definition for PCV10 or PCV13). Some sites may have used more than one product over time and may currently use a different one from that listed here. PCV13 is Pfizer's Prevnar13/Prevenar13; PCV10 is GSK's Synflorix. [2]All sites defined pneumococcus detected from CSF as pneumococcal meningitis. Listed here are any additional definitions of pneumococcal meningitis. CSF was not systematically collected in all meningitis cases across all sites. The sterile site was assumed to be blood for 8 sites that did not further specify. [3]Year of introduction as included in the analysis (year of introduction + 1 year if introduced in the last quarter of the year). If a site switched between PCV10 and PCV13, the year corresponds to the product with sufficient use to meet mature criteria. [4]Where available, the number following "PCR" indicates the number of serotypes able to be identified by PCR: PCR37 = real-time polymerase chain reaction (21 assays/37 serotypes): serotypes detected vary by regional scheme; PCR70 = conventional multiplex polymerase chain reaction (41

assays/70 serotypes); PCR35 = conventional multiplex polymerase chain reaction (35 serotypes); PCR38 = real-time polymerase chain reaction (serotypes detected by Africa scheme plus serotype 8); Q = Quellung reaction; L = Latex agglutination; WGS = Whole genome sequencing; NGS = Next generation sequencing; CST = Capsular sequence typing.

Reference

1. Cooper, D.; Yu, X.; Sidhu, M.; Nahm, M.H.; Fernsten, P.; Jansen, K.U. The 13-Valent Pneumococcal Conjugate Vaccine (PCV13) Elicits Cross-Functional Opsonophagocytic Killing Responses in Humans to Streptococcus Pneumoniae Serotypes 6C and 7A. *Vaccine* **2011**, *29*, 7207–7211, doi:10.1016/j.vaccine.2011.06.056.
